# Exploring Machine Learning Classification of Movement Phases in Hemiparetic Stroke Patients: A Controlled EEG-tDCS Study

**DOI:** 10.3390/brainsci15010028

**Published:** 2024-12-29

**Authors:** Rishishankar E. Suresh, M S Zobaer, Matthew J. Triano, Brian F. Saway, Parneet Grewal, Nathan C. Rowland

**Affiliations:** 1 College of Medicine, Medical University of South Carolina, Charleston, SC 29425, USA; sureshr@musc.edu (R.E.S.); triano@musc.edu (M.J.T.); saway@musc.edu (B.F.S.); rowlandn@musc.edu (N.C.R.); 2 MUSC Institute for Neuroscience Discovery (MIND), Medical University of South Carolina, Charleston, SC 29425, USA; grewalp@musc.edu; 3 Department of Neurosurgery, Medical University of South Carolina, Charleston, SC 29425, USA; 4 Department of Neurology, Medical University of South Carolina, Charleston, SC 29425, USA

**Keywords:** chronic stroke, machine learning, electroencephalogram, noninvasive brain stimulation, transcranial direct current stimulation

## Abstract

Background/Objectives: Noninvasive brain stimulation (NIBS) can boost motor recovery after a stroke. Certain movement phases are more responsive to NIBS, so a system that auto-detects these phases would optimize stimulation timing. This study assessed the effectiveness of various machine learning models in identifying movement phases in hemiparetic individuals undergoing simultaneous NIBS and EEG recordings. We hypothesized that transcranial direct current stimulation (tDCS), a form of NIBS, would enhance EEG signals related to movement phases and improve classification accuracy compared to sham stimulation. Methods: EEG data from 10 chronic stroke patients and 11 healthy controls were recorded before, during, and after tDCS. Eight machine learning algorithms and five ensemble methods were used to classify two movement phases (hold posture and reaching) during each of these periods. Data preprocessing included z-score normalization and frequency band power binning. Results: In chronic stroke participants who received active tDCS, the classification accuracy for hold vs. reach phases increased from pre-stimulation to the late intra-stimulation period (72.2% to 75.2%, *p* < 0.0001). Late active tDCS surpassed late sham tDCS classification (75.2% vs. 71.5%, *p* < 0.0001). Linear discriminant analysis was the most accurate (74.6%) algorithm with the shortest training time (0.9 s). Among ensemble methods, low gamma frequency (30–50 Hz) achieved the highest accuracy (74.5%), although this result did not achieve statistical significance for actively stimulated chronic stroke participants. Conclusions: Machine learning algorithms showed enhanced movement phase classification during active tDCS in chronic stroke participants. These results suggest their feasibility for real-time movement detection in neurorehabilitation, including brain–computer interfaces for stroke recovery.

## 1. Introduction

Chronic stroke affects over 10 million people in the US and remains a major source of disability worldwide [1,2]. Brain–computer interfaces (BCIs) are a suggested method to improve the quality of life for affected individuals given their potential to detect stroke severity, sense ongoing motor behaviors, and assist with longitudinal recovery [3,4,5,6,7,8]. To advance BCIs for chronic stroke towards clinical practice, many groups are interested in creating a simple BCI embedded with machine learning (ML) that can be deployed at the population scale [9]. Yet it is currently unknown what neural input features and ML approaches are optimized for this task.

Electroencephalography (EEG) recordings are a noninvasive and an accessible method of sampling brain activity for neural computations in BCI use. Moreover, EEG can be paired with noninvasive brain stimulation (NIBS) to enhance certain features of control signal classification [10]. Transcranial direct current stimulation (tDCS), a form of NIBS, has been used in stroke rehabilitation [11] and has the specific advantage of continuous and simultaneous stimulation and EEG recording [10,12]. Anodal tDCS applied to the motor cortex has also been shown to affect BCI performance [12]. While mental rehearsal has evolved into the standard paradigm for BCI studies [13,14,15,16,17], particularly in those with tetraplegia, the vast majority of participants with stroke retain some level of motor capability. Accordingly, many have questioned whether ML-assisted EEG classification in stroke participants performing real movements requires the modeling of a substantively different parameter space [18,19,20]. As an example, Mebarkia et al. [21] found that layering three support vector machines (SVMs) (i.e., multi-voting) was necessary to exceed 90% accuracy in classifying left versus right hand movements in 3D space; however, this BCI architecture has only been tested in healthy participants.

The parameter space for EEG BCIs designed for stroke rehabilitation is extensive. First, the spectral content of EEG recordings in healthy controls is significantly different from that of individuals post-stroke [22,23,24]. For instance, mu (8–12 Hz) and beta (13–30 Hz) power are attenuated following a stroke, yet event-related desynchronization (ERD) and synchronization (ERS) of mu and beta power are frequently used to provide the feedback signal for decoding movement intention [25,26,27]. For those with severe motor deficits, ERD/ERS can be effectively absent, and thus, alternate control signals must be identified, though it is not clear what approach should be used to select surrogate markers. Second, vascular compromise gives rise to hemispheric asymmetry (i.e., ipsi- vs. contralesional), and this asymmetry is reflected as imbalanced oscillatory patterns following stroke [28,29,30]. Whether these factors preclude ML classification of brain states during real movement is a critical question to address prior to the development of a clinically accepted BCI for post-stroke hemiparesis.

In this study, we aimed to test the overall hypothesis that EEG data recorded during different movement phases in individuals with chronic stroke can be accurately classified using ML. We further explored whether transcranial direct current stimulation (tDCS), which can be activated continuously during EEG recording, boosts the ML classification accuracy for certain movement phases compared to sham stimulation. As a control, we employed an identical ML pipeline in healthy participants.

## 2. Materials and Methods

### 2.1. Sample Size Calculation

In a previous study comparing Parkinson’s (n = 10) and essential tremor (n = 8) patients, we observed an absolute difference in cortical beta event-related desynchronization (ERD) during movement preparation of 0.24 with a standard deviation of 0.19 (PD −0.31 ± 0.06, ET −0.071 ± 0.05, log10(μV)^2^/Hz, mean ± SE, *p* = 0.0061) [31]. Based on this experience, with an observed absolute difference in cortical beta ERD of 0.24 log10(μV)^2^/Hz and an assumed standard deviation to be as observed in our preliminary data for the PD group, we estimated that a sample size of 20 subjects for both the HC and CS groups would be sufficient to yield a power of 91% assuming a moderate intra-class correlation of 0.7. The sample size was divided in half for this pilot study, which was focused on the feasibility of the methods, with plans to carry out a fully powered study as an immediate follow-up to this manuscript.

### 2.2. Participants

**Figure 1:**
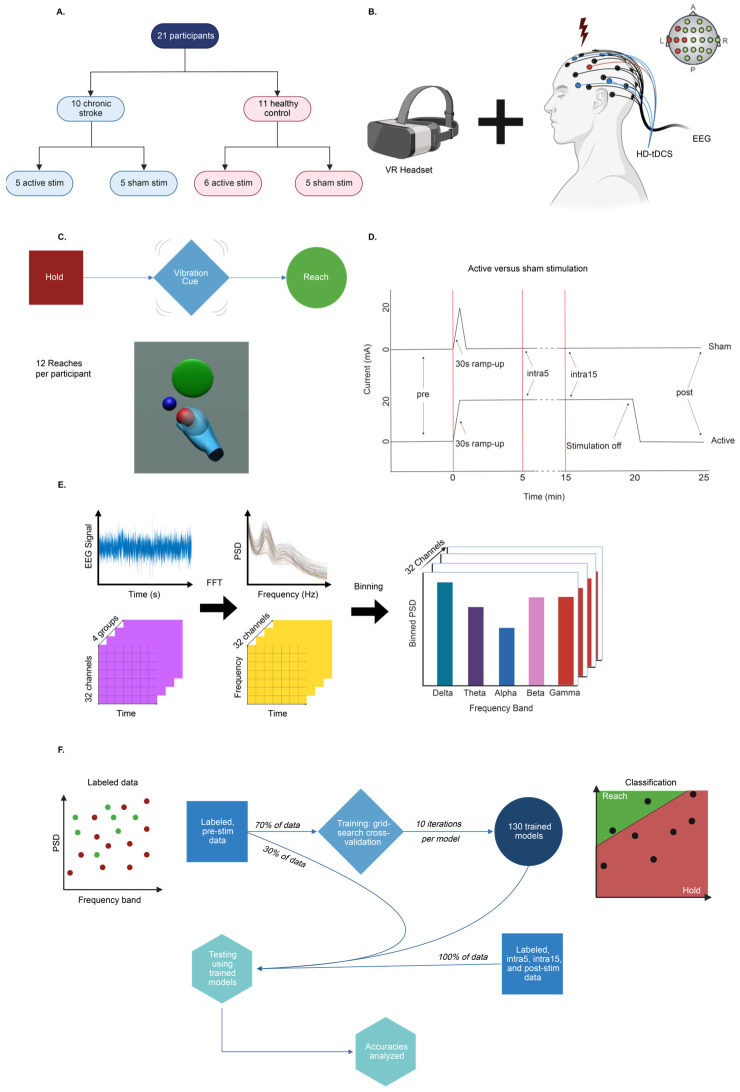
(**A**) A total of 10 participants with chronic stroke and 11 healthy controls were randomized into active and sham stimulation groups. (**B**) The participants were fitted with EEG electrodes and HD-tDCS delivering anodal stimulation to the ipsilesional side (contralateral to the motor deficit); for healthy participants, laterality was randomized. (**C**) During the stimulation phase, the participants performed a VR motor task involving reaches toward a virtual blue sphere target (3 cm radius) placed 0.3–0.5 m away. The task had three steps: hold, prep (Cue), and move. An example VR scene is shown. (**D**) Sham participants received 30 s of ramp-up current, then no stimulation, while active stimulation included 30 s ramp-up followed by 20 min of stimulation. Twelve reaches were performed at each time period. EEG was recorded at pre, 5 min (intra5), 15 min (intra15), and post-stimulation (post). (**E**) The raw EEG signal was recorded from all channels. After normalization, the power spectral density was calculated and binned across frequency bands to complete feature extraction. (**F**) Thirteen ML models were trained using 70% of the pre-stim data and then tested on 30% of the pre-stim data and 100% of the data from other time periods. This was performed to simulate ex vivo training of an onboard BCI.

Ten chronic stroke (CS) participants with upper-extremity hemiparesis and eleven healthy controls (HCs) were included in this study, which was approved by the Medical University of South Carolina Institutional Review Board (Pro#00087153; date: 16 July 2022) (Figure 1A). Hemiparetic patients were chosen as the vast majority of patients with chronic stroke retain some degree of motor function. Chronic stroke was defined as greater than six months from a sentinel infarct as determined by a fellowship-trained stroke neurologist (PG). Only participants who could raise and reach with the affected upper extremity were included. Participants with a history of seizure, intracranial mass, infection or cerebrovascular malformation, cranial defect, radiotherapy to the brain, scalp injury or disease, pregnancy, or the inability to consent to the study were excluded. The mean time after stroke for CS participants was 98.8 ± 36.7 months. The CS and HC groups did not differ with regard to sex (χ^2^_[1]_ < 0; *p* > 0.05). The mean age was higher in CS (63.3 ± 10.2 years; range = 30 to 78 years) than HC participants (46.3 ± 11.3 years; range = 24 to 73 years, t_[18]_ = 2.31, *p* = 0.0319). Six CS participants had left-sided infarcts, while four had right-sided infarcts.

### 2.3. Randomization and Single Blinding

Study participants in each CS or HC cohort were assigned to either a stimulation or sham group (Figure 1A). Each participant was assigned to the opposite group (i.e., stimulation or sham) as the previously recruited participant to maintain balance. Participants were blinded to their treatment assignment. For the CS cohort, 5 participants were assigned to active stim, and 5 were assigned to sham; for the HC cohort, 6 were assigned to active stim, and 5 were assigned to sham (Figure 1A). The sham group underwent a placebo procedure, in which current was applied in increasing steps for 30 s to induce a tingling sensation in the scalp. This mimicked the procedure in the stimulation group that involved an identical 30 s of current ramp before applying 20 min of DC current. Research staff operating the tDCS device were aware of the group assignment. Randomization and blinding were applied to both CS and HC cohorts in an identical fashion.

### 2.4. Task and Dataset

During a single recording session, the participants were fitted with a total of 20 Ag-AgCl EEG electrodes using the 10–20 International EEG system (DC115, Rhythmlink International LLC, Columbia, SC, USA) (Figure 1B). Next, a 5-electrode, center-surround, anodal high-density (HD) transcranial direct current stimulation (tDCS) montage was arranged on the scalp contralateral to the affected arm (model 2001tE and 4×1-C3ASKU 4×1-C3ASoterix Medical; 4×1 HD-tDCS/HD-tES adaptor, Soterix Medical, Inc, Woodbridge, NJ, USA). The central anodal tDCS electrode was positioned near the C3 or C4 EEG electrode, depending on the laterality of the upper extremity performing the task. The C3 and C4 electrodes represent the site of the primary motor cortex (M1) ipsilateral to the stroke lesion for participants with stroke. The surrounding cathodal tDCS electrodes were arranged in a square configuration in relation to the central anode. All tDCS electrodes were placed adjacent to, but out of direct contact with, nearby EEG electrodes (Figure 1B). Next, an Oculus Rift (Model C4-A, Menlo Park, CA, USA) virtual reality (VR) headset was carefully fitted over the EEG-tDCS arrangement, and a wireless controller containing an accelerometer was placed in the affected hand. For healthy participants, the hand used for the task was randomized. EEG signals were sampled at 1024 Hz (Natus^®^ Neuroworks^®^, Pleasanton, CA, USA). An arm reaching task designed in the Unity^®^ (version 2022.2, Unity Technologies, San Francisco, CA, USA) programming environment began with a holding position requiring a slightly outstretched upper limb to remain in place for several seconds prior to the appearance of a colored sphere. Once the sphere appeared, a reaching movement was made by the participant to virtually “touch” the sphere and return to the holding position (the total hand distance traveled ranged from 15 to 50 cm). The duration of the holding position was randomly varied between 2 and 5 s. Additionally, the location of the sphere was randomly varied between different locations within the virtual environment to reduce learning of the task over time. In between the hold and reach periods, a 0.5 s preparatory (or “prep”) cue was delivered in the form of a vibratory pulse of the controller. The participants were not explicitly instructed to attend to this cue. The trial ended after 12 consecutive hold, prep, and reach cycles were completed (the average trial duration for all 12 reaches was approximately 3 min) (Figure 1C). The number of trials was selected empirically to protect against fatigue for mild-to-moderate hemiparetic individuals. For analysis purposes, each movement cycle was divided into the following states, or “epochs”: the hold epoch, occurring prior to the prep cue, and the reach epoch occurring from initiation of movement until returning to the holding position. After an initial trial during the pre-stimulation period, the tDCS system was activated and within 30 s reached a maximum current delivery of 2.0 mA. Two additional trials of 12 reach cycles were performed at 5 and 15 min each after tDCS activation. Next, at 20 min after tDCS activation, the current was switched off. Five minutes after the deactivation of tDCS, a fourth and final trial was performed (Figure 1D). Thus, all participants performed a total of four trials of 12 reaches each. As per the published tDCS literature [10,32,33], the participants in the sham control group had tDCS activated for 30 s before ramping down to zero over an additional 30 s. This short period of stimulation mimics the 30 s of scalp tingling induced by active tDCS [33]. The HC group underwent the same randomization and procedures. The entire experiment for each participant lasted approximately one hour. The participants were given the option to take breaks to prevent fatigue. VR and EEG signals were labeled using synchronized TTL pulses across all data streams.

### 2.5. Preprocessing and Feature Extraction

Depending on the configuration, BCIs for stroke may have low computational power, which limits access to other critical signals such as electrooculography (EOG) and electromyography (EMG). An additional constraint observed by Winkler et al. and McDermott et al. [34,35] is that artifact rejection can attenuate BCI performance due to signal loss. Thus, we omitted resampling and limited signal preprocessing to bandpass filtering (a 1 Hz high-pass filter and 50 Hz low-pass filter were employed to remove environmental noise [36] and a portion of EOG and EMG artifacts, respectively [37]). All data were z-score transformed over the entire EEG signal for each channel per participant. Normalization was performed via the method shown in Equation (1), where x, $\bar{x}$, and σ represent the raw, mean, and standard deviation of the input values [38].

(1) $$x_{norm}=\frac{x-\bar{x}}{\sigma }$$

Recordings were divided into 1 s epochs to balance point-to-point variations in the signal while maintaining adequate temporal resolution. Power spectral densities (PSDs) were calculated for each epoch using Welch’s method, which applies the discrete Fourier transform (DFT) to several contiguous windowed subsets of the original signal [39]. Hann windowing was used to generate windowed segments with 50% overlap, and the number of Fast Fourier Transform (FFT) segments was set to the sample rate to maintain a spectral resolution of 1 Hz, as described above (Figure 1E). PSD values were subsequently resolved into the following frequency bands: delta (1–4 Hz), theta (4–8 Hz), alpha (8–12 Hz), beta (12–30 Hz), and gamma (30–50 Hz). As the bands did not exceed 50 Hz, a 60 Hz notch filter was not applied.

Extracted features from all participants in each of the four experimental groups (i.e., CS active stimulation, CS sham stimulation, HC active stimulation, and HC sham stimulation) were compiled into one dataset per group for model training. The labeled dataset in each experimental group was split 70:30 for model training and testing with grid search cross-validation used for hyperparameter tuning. The model training dataset consisted of 5100 samples with feature dimensions of (6, 5100), including 5 frequency band features and 1 electrode feature; the model testing dataset contained 2300 samples with dimensions of (6, 2300). Model training consisted of the classification of extracted features into 2 pre-labeled classes: the “hold” phase, during which participants were stationary, and the “reach” phase, during which participants extended their affected or dominant hand towards a VR target. Model training was carried out 10 times for each model per trial, and the average training times, with standard deviations, were recorded. Thus, models were trained for each experimental group rather than individual participants. From this dataset, a total of 4030 models were trained with a total computing time of 103 h. Training was performed on a 6th Gen Intel Xeon (R) Gold 6226R processor at 2.90 GHz with 64 cores and 187.5 GB RAM in serial processing alongside two NVIDIA RTX A5000 GPUs. Trained models were then tested on the remaining 30% of pre-stimulation data to obtain accuracies for the pre-stimulation time period. For subsequent time periods, 100% of the data were classified using these pre-trained models without further training, and these are the accuracies reported in the text (Figure 1F). All analyses were conducted in Python 3.9 using the NumPy [40], SciPy [40], and Scikit-Learn libraries [41].

### 2.6. Machine Learning Implementation

The classification accuracy of movement phases was tested among 13 different machine learning algorithms on the same dataset. These were selected to explore the effect of the disease state, stimulation state, time period, frequency band, and EEG electrode location. The time periods examined were pre-stimulation, intra-stimulation at 5 and 15 min after stimulation began, and post-stimulation, hereafter referred to as “Pre”, “Intra5”, “Intra15”, and “Post”. Features were tested in limited combinations to (1) reduce the exponential increase in model permutations and (2) to narrow the parameter space with a focus on clinical interpretability. A set of 8 primary algorithms was selected to identify an optimal model with respect to accuracy, computational demand, and training time. Additionally, we created 5 ensemble models using these 8 primary algorithms with varied weights to assess their combined accuracy. The following algorithms were chosen for this study: logistic regression (LR), linear discriminant analysis (LDA), decision trees (DT), Naïve Bayes (NB), K-nearest neighbors (KNN), random forest (RF), AdaBoost, and XGBoost.

LR was chosen due to its prior use in motor imagery classification derived from EEG signals [42]. LDA classifiers model the distribution of each class and were included due to their ability to perform dimensionality reduction and minimize the training time [43]. DT and RF were chosen to detect complex patterns in binarized data that may lead to a higher classification accuracy. The Naïve Bayes classifier applies Bayes’ theorem to calculate the probability of an observation belonging to a given class based on the assumption that the data are distributed in a Gaussian manner; NB was included due to its minimal training time and mathematical simplicity [43]. KNN was included to determine if the movement phases exhibited clustering behavior in the associated feature space, as this would reveal insights beyond improved classification accuracy. The boosting algorithms XGboost and AdaBoost were included to detect the importance of incorrectly classified data points.

The hyperparameter search process for each classifier was defined in the following way: For LR, a univariate grid search was performed on the parameter C for values 2x for 15 ≤ x < 35. For LDA, comparisons were conducted for accuracies achieved by singular value decomposition (SVD), least squares (LSQR), and eigen solvers. Although all models demonstrated similar accuracies, SVD was chosen since it does not compute the covariance matrix and, therefore, has a shorter run time. For NB classification, a Gaussian implementation was used due to the Gaussian nature of epoched EEG data. A parameter representing the variance was computed using 10x for −15 ≤ x < 0. For KNN, the number of neighbors considered was varied for 3 ≤ x < 10. For DT, the minimum weight fraction of each leaf node was empirically determined to be 0. A grid search was performed to optimize the minimum number of samples required to split individual nodes (varied for 2 ≤ x < 11) and the maximum depth allowed for trees (varied for 2 ≤ x < 30). For RF, the minimum number of samples per leaf was optimally determined to be 1, the method of determining the maximum samples to split a node was set to the square root of the total number of samples, and the maximum depth of each tree was set to 30. A grid search was performed to optimize the minimum number of samples required to split individual nodes (varied from 2 ≤ x < 5); the number of individual trees was varied over the set 5n for 5 ≤ n < 21. Feature importance was calculated by taking each feature’s average depth of use and weighing the average from one relative to the other features’ depths. The earlier a feature was used in a tree, the more important it was considered. For AdaBoost, NB and DT were contrasted as base estimators, and the number of individual estimators was varied by 25x for 8 ≤ x ≤ 16. For XGBoost, the number of individual estimators was varied by 25x for 50 ≤ x ≤ 400, and the max tree depth varied by 5x for 5 ≤ x ≤ 30. The “Hist” method, as implemented by XGBoost 2.0.3 [44], was chosen for the Tree Method hyperparameter to reduce the training time. For voting classifiers, an ensemble of pre-trained models for LR, LDA, DT, RF, NB, and KNN was first created. One hard-voting classifier and four soft-voting classifiers among these were then utilized. Soft-voting classifiers used the following weight methods: uniform weights (termed “uni”), weights determined by the individual models’ training set accuracy (termed “train”), uniform weights determined by the highest model accuracy (termed “hard”), and weights predetermined based upon the empirical global accuracy of all models within the ensemble (termed “global”). The ensemble labeled “me” was weighted based on the mean of several selected base estimators that appeared to perform better during the initial classification tests used to classify hold vs. reach. Effect weights, hyperparameters, and computation times were saved for each training. Prediction results were stored as text files labeled by the electrode and feature. Note that all accuracies depicted are the classification accuracies of the validation (i.e., testing) set; no samples in the validation set were used to train any of the models.

### 2.7. Statistical Analysis

Statistical analysis was conducted using the rstatix package for R (version 4.3.1) [45]. Combining data using two to three features for each model (e.g., grouping all electrodes and frequencies) resulted in groups sufficiently large to satisfy the central limit theorem, which were, therefore, treated as parametric data. For comparisons between multiple groups, student’s t-test and repeated measures ANOVA (one and two way) were used. Sphericity was tested using Mauchly’s test and, when violated, was corrected using the Greenhouse–Geisser correction. Bonferroni adjustment was applied for multiple comparisons. Specific comparisons are highlighted in the text with the appropriate test statistics; full results are given Supplementary Table S6. Data visualizations were generated using the ggpubr package for R [46]. The default threshold for significance was set at *p* < 0.05 for all tests. All error bars represent the standard error of the mean. Effect sizes are reported in Supplementary Table S11.

## 3. Results

### 3.1. Baseline Classification Accuracies

**Figure 2:**
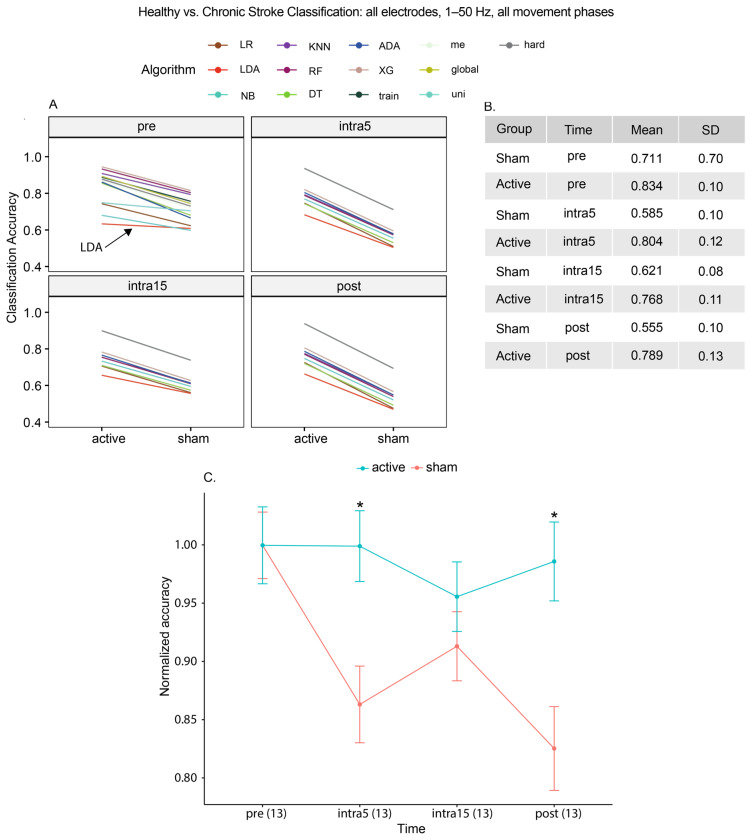
(**A**) Classification of disease state (healthy versus chronic stroke) using all frequencies from 1 to 50 Hz and all electrodes. Although most algorithms detected differences prior to stimulation, LDA was not affected. (**B**) Mean accuracies. (**C**) To account for baseline differences at the pre-stimulation time periods, we normalized the accuracies to the pre-stimulation accuracy for each group. Asterisks (*) indicate a significant difference between active and sham groups. We observed a significantly increased classification accuracy for the active stim group at the intra5 and post-stimulation time periods, with accuracies converging at intra15. Values in parentheses represent the number of algorithms per group at each time period.

To investigate the baseline classification characteristics in our cohort, we compared the accuracy of each algorithm in distinguishing healthy from chronic stroke participants. This baseline analysis was carried out using all electrodes, frequency bands, and movement phases grouped together. This is also an important criterion our models need to meet, since BCIs, like all neuromodulatory devices, are designed to be disease specific. During the pre-stimulation time period, we observed that the mean accuracy of classifying HC and CS participants was 83.4% for the active group and 71.1% for the sham group (t_[22.1]_ = −3.60, *p* = 0.0016, Figure 2A), suggesting that some baseline variation persisted between the active and sham groups after randomization. This difference was notable in 12 out of 13 algorithms, the exception being linear discriminant analysis (LDA). To account for this variation, we normalized the accuracies for each group using their respective pre-stimulation accuracy (Figure 2C), and the resulting curves showed wide deviations between the groups in the classification accuracy over time. As expected, a two-way repeated measures ANOVA revealed a statistically significant effect of the stimulation group as a function of accuracy (F_[1,24]_ = 4.502, *p* = 0.044, see Supplementary Table S6 for full statistical results, n.b., red italicized text). Notably, normalized classification accuracies between the groups were most different at the post-stimulation state (active: 98.6%, sham: 82.5%, t_[23.8]_ = −3.25, *p* = 0.0013). These results confirm that differences in the classification of disease states between active and sham tDCS are statistically significant, consistent with our overall hypothesis.

### 3.2. Movement Phase

**Figure 3:**
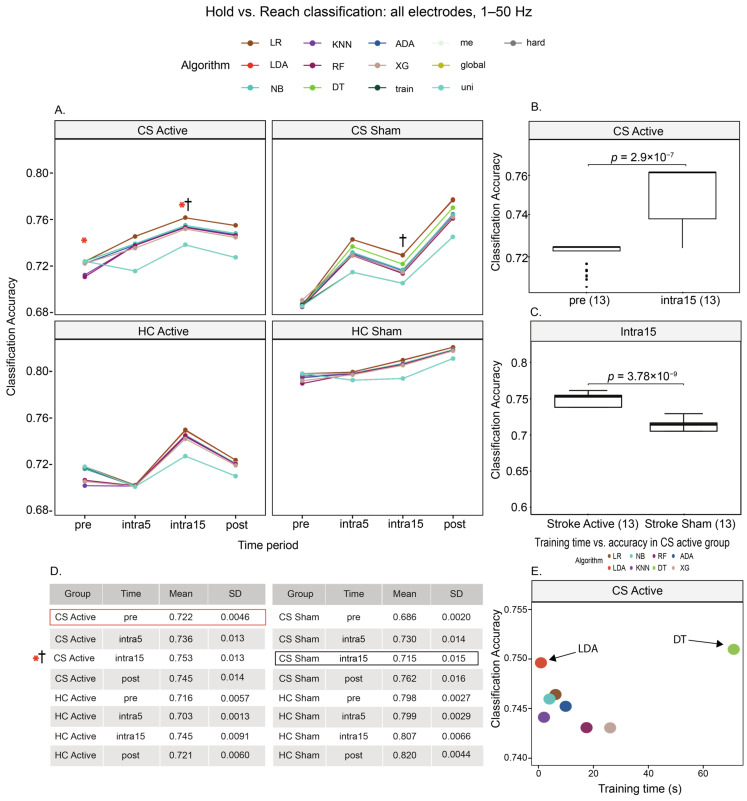
(**A**) Hold versus reach movement classification using all frequencies of 1–50 Hz and all electrodes. In the CS active group, the classification accuracy increased after stimulation and peaked at intra15 (*, asterisk). Furthermore, the accuracy at this time period was significantly higher than in the CS sham group at the same time (†, cross). (**B**) Movement classification was higher at intra15 than pre in the CS active group. (**C**) Movement classification was higher for intra15 CS active than intra15 CS sham. Values in parentheses represent the number of algorithms per group at each time period. (**D**) Mean accuracies. Values in parentheses represent the number of algorithms per group at each time period. (**E**) We observed a tradeoff between the training time and accuracy, as LDA produced the highest accuracy with a short training time compared to the other models with the exception of DT, which obtained a slightly higher mean accuracy but required the longest training time.

To investigate the accuracy of each algorithm in discriminating hold vs. reach movement phases, we trained ML models using all electrodes and frequency bands separated by disease and stimulation state over time (Figure 3). We observed that the mean accuracy of classifying hold vs. reach was 72.2% for the CS active group, 68.6% for the CS sham group, 71.6% for the HC active group, and 79.6% for the HC sham group at the pre-stimulation time period. For the CS active group, the mean classification accuracy increased during the early and late stimulation periods, peaking at intra15 (pre-stim: 72.2% vs. intra15: 75.3%, t_[17.1]_ = 9.20, *p* < 0.0001, Figure 3A,B, see Supplementary Table S6). Additionally, the mean classification accuracy between hold vs. reach was higher at intra15 for the CS active versus the CS sham cohort (active: 75.3%, sham: 71.5%, t_[23.7]_ = −9.73, *p* < 0.0001, Figure 3C). A two-way repeated measures ANOVA confirmed a significant effect on the accuracy as a function of the stimulation group (F_[1,24]_ = 20.174, *p* = 0.00015), as well as a significant effect of time (F_[3,72]_ = 234.59, *p* < 0.0001) and group–time interaction (F_[3,72]_ = 111.599, *p* < 0.0001). These results suggest divergence in the classification accuracy as a function of active tDCS while controlling for the disease state. Interestingly, the mean classification accuracy between hold vs. reach was significantly different at all time periods except intra15 for the CS active versus the HC active cohort (CS active: 75.3%, HC active: 74.5%, t_[24]_ = 2.27, *p* = 0.1, see Supplementary Table S6). Thus, late active tDCS exacerbates movement phase classification differences with sham tDCS (i.e., stimulation state) and attenuates classification differences between healthy and stroke participants (i.e., disease state).

In the CS active group, LDA performed hold vs. reach classification with a mean accuracy of 74.6% at the shortest mean training time of 0.9 s per iteration. By comparison, DT performed hold vs. reach classification with a 75.1% accuracy at the longest average training time of 1 min 11.1 s per model.

### 3.3. Frequency Band

**Figure 4:**
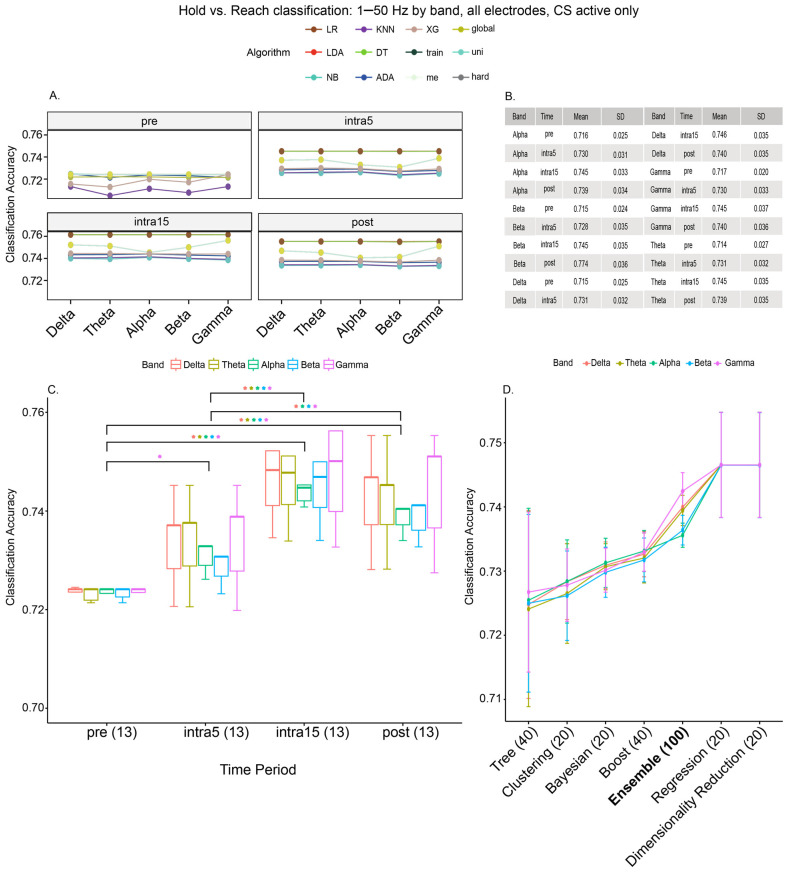
(**A**) Hold versus reach movement classification using all frequencies of 1–50 Hz and all electrodes by the frequency band used (delta through gamma) in CS active participants only. Gamma PSD alone produced the highest classification accuracy for most models, although this was not statistically significant. (**B**) Mean accuracies. (**C**) When comparing the classification accuracy over time by band, we observe a broadband increase in the accuracy, seen in all bands at intra15. We also observe some differences in the band response, highlighted here using colored asterisks corresponding to each band. Values in parentheses represent the number of algorithms per individual frequency band at each time period. (**D**) When aggregating algorithms by method, we observed that ensemble methods (such as global voting, or hard voting) resolved frequency bands more so than other models, although this was not significant. Dimensionality reduction (LDA) and regression (LR) methods outperformed the others. Values in parentheses represent the number of algorithms per method at each time period.

To investigate the accuracy of each algorithm in discriminating hold vs. reach phases by frequency band, we created ML models using all electrodes recorded in the CS active group only (Figure 4). We observed that the mean accuracy of classifying hold vs. reach was consistently higher in the stimulation and post-stimulation time periods for all frequency bands (Figure 4A,C; note that for the pre-stimulation period, the RF classification accuracy was below 65% for all frequency bands and is not shown in Figure 4A,C). When examining individual frequencies, only a low gamma classification accuracy significantly increased immediately after stimulation (gamma: pre = 71.7%, intra5 = 73.5%, t_[15.9]_ = −2.88, *p* = 0.03, Figure 4C; outliers omitted for clarity), although there is a broadband accuracy increase at intra15 observed for all bands when compared to pre (Figure 4C, with multiple comparison results in Supplementary Table S6). This increased accuracy persisted into the post-stimulation period (Figure 4C, with multiple comparison results in Supplementary Table S6). Interestingly, the largest differences in the classification accuracies by frequency band appeared between ML approaches rather than individual algorithms (Figure 4D, F_[6,253]_ = 6.304, *p* < 0.0001, Supplementary Table S6). While a two-way ANOVA between the ML method and frequency band across all time periods confirmed a significant effect on the accuracy as a function of the ML method (F_[6,225]_ = 5.564, *p* < 0.0001), it uncovered no significant effect of the frequency band (F_[4,225]_ = 0.203, *p* = 0.936) or method–frequency band interaction (F_[24,225]_ = 0.046, *p* = 1.00). Notwithstanding, we observed a sub-significant increase in the low gamma frequency band resolution, being most prominent in the ensemble methods (F_[4,95]_ = 1.353, *p* = 0.256, Figure 4D), whereas other methods appeared relatively indifferent to the frequency band choice.

### 3.4. Electrode Laterality

**Figure 5:**
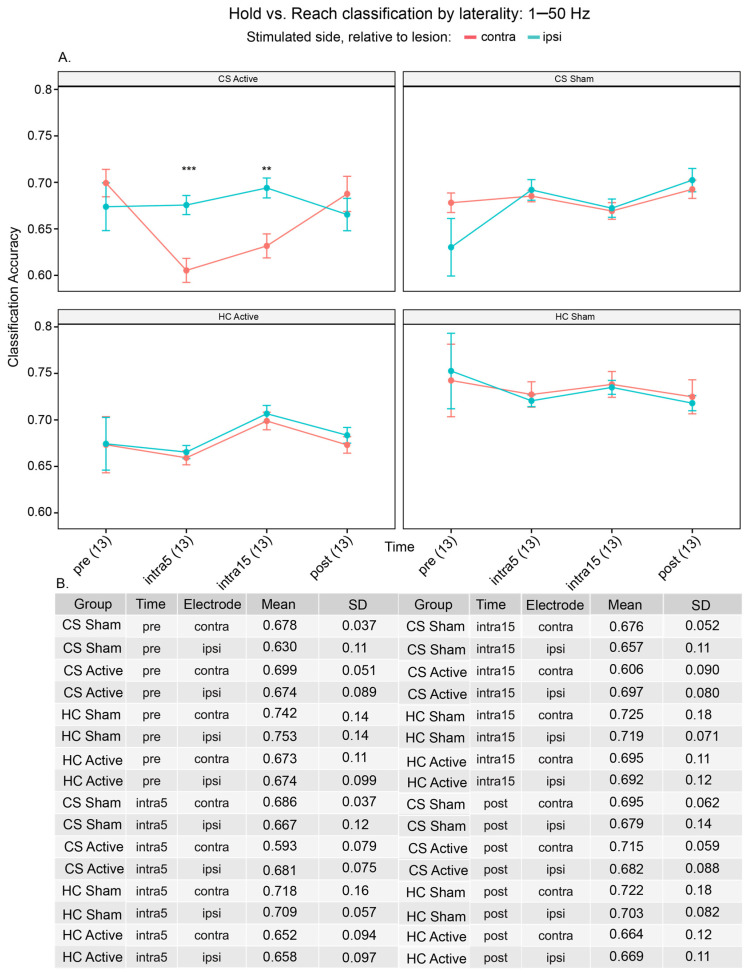
(**A**) Hold versus reach movement classification using C3 or C4 electrodes and all frequencies of 1–50 Hz. Number of asterisks (*) represents significance level between contra- and ipsi-lesional accuracy. Here, we investigated the effect of recording electrode lesion laterality. We observed that contralesional classification accuracy significantly decreases during stimulation compared to ipsilesional accuracy in CS active participants; this is not seen in any of the other groups. Values in parentheses represent the number of algorithms per group at each time period. (**B**) Mean accuracies.

To investigate the accuracy of each algorithm in discriminating hold vs. reach phases by electrode laterality, we created ML models using all frequency bands grouped together. We labeled the electrode overlying the motor cortex (C3 or C4) contralateral to the hand used to perform the task as the ipsi-stimulated electrode. That is, if the right hand performed the task, then the C3 electrode was labeled as the ipsi-stimulated electrode while the C4 electrode was labeled as the contra-stimulated electrode and vice versa. We observed that the mean accuracy of classifying hold vs. reach was consistently higher in the contra-stimulated electrode for the CS sham and HC sham groups), with the exception of the pre-stimulation period in the HC sham cohort. In contrast, in the CS active group, the classification accuracy was highest in the ipsi-stimulated electrode during active stimulation periods only (intra5: contra = 59.3%, ipsi = 68.1%, t_[12]_ = −7.01, *p* = 5.64 × 10^−5^; intra15: contra = 60.6%, ipsi = 69.7%, t_[12]_ = −4.71, *p* = 0.00204, Figure 5A, see Supplementary Table S6). As expected, a two-way repeated measures ANOVA identified a significant effect of time (F_[3,72]_ = 4.206, *p* = 0.032) but not the electrode group (F_[1,24]_ = 1.887, *p* = 0.182), although a significant interaction between the electrode and time existed (F_[3,72]_ = 7.196, *p* = 0.005).

Notably, the classification accuracy was similar in both the contra-stimulated and ipsi-stimulated electrodes in the HC active group, suggesting that the higher accuracy in the ipsi-stimulated electrode in the CS active group is not likely to be driven by stimulation artifacts. Moreover, the classification accuracy in the ipsi-stimulated electrode in the CS active group is constant relative to the pre-stimulation state, again suggesting a physiological response to stimulation that peaks at the intra15 time period (Figure 5A, Supplementary Tables S5 and S6). Overall, the classification accuracy for each electrode is dependent on the time of stimulation, i.e., whether stimulation is on or off.

## 4. Discussion

As neuromodulation becomes an accepted adjunct for chronic stroke recovery, the potential use of NIBS to assist in autonomously identifying movement phases from brain recordings has become a topic of great interest. The detection of movement intention alone using EEG recordings has been successfully performed in several studies using healthy and tetraplegic participants [26,47,48]. However, real movement classification in individuals with hemiparesis, the largest group of chronic stroke patients, is not well understood, including the dimensionality involved in modeling relevant parameters. Moreover, in implanted brain recording and stimulation systems, computation power is limited to the onboard processor, which prohibits the typical types of algorithms employed in these studies, e.g., deep learning and other neural network-based strategies. To surpass these constraints, using minimal signal preprocessing, we explored supervised ML modeling of EEG recordings in order to understand the range of accuracies and modeling times for potential use as control signal classifiers. The central problem addressed in this study is whether movement classification based on EEG recordings is plausible (i.e., above chance) in chronic stroke survivors undergoing NIBS during the performance of a functional task. Our overall finding is that EEG data recorded during different movement phases in individuals with chronic stroke can be accurately classified using ML. Additionally, tDCS improved classification accuracy primarily in the chronic stroke active stimulation group, with a slight preference for gamma frequency bands using the ensemble methods.

Comparing our results to similar studies that developed EEG classifiers for movement phases, SVMs performed modestly better in 46.4% of models than LDA and LR in classifying rest, simple arm movements, goal-oriented arm movements, and hand clenching during motor imagery [43]. In our study, the performance of LDA for hold vs. reach (at 74.5% accuracy during the intra15 time period) was within range of the accuracies reported by Yong and Menon [43] (75–81% accuracy) and higher than those of Rodrigo et al. [42] (64–68% accuracy). The voting classifiers trained in this study for hold vs. reach in the chronic stroke active stimulation cohort (at the intra15 time period, see Supplementary Table S3) achieved an accuracy of 73.8%, which is not as strong as the voting classifier created by Khrishna et al. [49] (86% accuracy). Similar to the dataset used by Mebarkia and Reffad [21], the dataset used by Khrishna et al. [49] classified motor imagery in the right arm, left arm, right foot, and left foot without any consideration for holding or preparation, which may explain the stronger performance. The AdaBoost classifier trained as part of our study achieved an accuracy of 75.3% using decision trees as the classifier base, which is comparable to the AdaBoost classifiers trained by Gao et al. [50], who used SVM and LDA bases to achieve accuracies of 74% and 72%, respectively.

A few studies have combined tDCS and BCI devices with mixed results. tDCS alters the brain’s electric field, modulating ionic currents and neuronal membrane potentials to enhance synaptic plasticity [33,51]. These induced changes are hypothesized to improve information flow across neural networks, analogous to optimizing resonance frequencies in coupled oscillatory systems for greater synchronization and efficiency [51]. Matsumoto et al. [52]. used a motor imagery (MI) BCI in concert with multiple 1 mA 10 min tDCS sessions in six healthy participants. In their investigation, mu ERD improved with anodal and attenuated with cathodal stimulation [52]. Kasashima and colleagues [12] repeated this paradigm in participants with post-stroke hemiparesis, demonstrating similar results. In a study by Wei et al. [53], tDCS specifically modulated upper mu (10–14 Hz) and beta (14–26 Hz) frequencies in 32 healthy controls. Hong and colleagues [54] introduced diffusion and perfusion MRI following tDCS in combination with an MI-BCI. Tractography estimates showed significant changes on the ipsilesional side for participants receiving tDCS, although no difference in motor improvement was observed between active and sham groups [54]. Overall, tDCS did not appear to influence MI-BCI performance in a randomized, double-blinded controlled trial in 19 participants with stroke [10]. These results did not differ from a subsequent study in which functional MRI was used to derive a low-frequency fluctuation metric [32]. None of the studies outlined utilized real movements as a control.

Although we observed that the ensemble methods achieved the highest hold vs. reach classification using gamma power as a feature, the modulation of gamma power in individuals post-stroke is only sparsely reported. In a study by Tecchio et al. [55], increased gamma power (33.5–44 Hz) in the affected hemisphere of chronic stroke participants was correlated with motor improvement using magnetoencephalography recordings. Moreover, Pellegrino and colleagues [56] demonstrated that gamma reactivity to an auditory stimulus in chronic stroke participants was tightly correlated to the clinical outcome, as measured using the Barthel Index and Functional Independence Measure. Yet in a recent systematic analysis of randomized controlled trials examining the utility of BCIs in stroke motor recovery, gamma power was absent from the frequency bands investigated as a potential biomarker [7].

### Limitations

This study was not without limitations. First, a small sample size limits the interpretability of our classification results. Notwithstanding, Bolognini et al. [57] performed a similar exploratory study evaluating the interaction between tDCS and constraint-induced movement therapy in chronic stroke and reported significant complementary effects using only 14 participants. Similarly, Biasucci et al. [58] performed a sham-controlled study evaluating the effect of BCI-coupled functional electric stimulation (FES) on motor outcomes and reported significant improvement when compared to sham, with only 27 participants and 14 in the experimental arm. These and other similar studies with limited participant numbers are reported in Supplementary Table S9. A limited sample size further predisposes to baseline differences between groups, as is demonstrated by the discrepancy in the classification accuracy between our pre-stimulation stroke cohorts. This group heterogeneity is further exacerbated by the sensitivity of machine learning models to cross-validation folds, which are randomly generated. In this situation, Watson and Holmes describe the use of a subgroup-specific statistical analysis plan for evaluation; however, this is impractical a priori in a single-subject chronic stroke BCI; furthermore, the use of modifications to machine learning models, as described in that study, may require computational resources and time that may not be available to BCI devices [59]. Other tDCS studies have employed a crossover design to mitigate this heterogeneity to some success [60,61], although this approach risks carryover effects from tDCS contaminating the sham phase, as was noted by Klomjai et al. [60]. We have attempted to address this using repeated measures, i.e., within-subject statistical design. Significant differences in the mean age between our chronic stroke and healthy cohorts were also observed, potentially confounding observed differences in the classification accuracy that may have been affected by age-related changes in the brain.

To attempt to mimic the processing and bit-rate constraints of a fully implantable BCI system [62], we avoided training more comprehensive deep learning models. Nevertheless, a robust classification pipeline using Convolutional Neural Networks was described by Lun et al. [63], who trained a five-layer model on the Physionet database [64,65]. Remarkably, using only 10 participants from that dataset, a global accuracy of 94% or above was demonstrated. Similar to our study, they limited the pre-processing of the EEG signal and still achieved high classification accuracies [63]. In our study, training was limited to twelve repetitions per task and four tasks per participant during a single one-hour experimental session. Nevertheless, our results are almost certainly confounded by some proportion of learning of the task, although we attempted to limit this by randomizing several features, including the time between movement phases and the location of reach target. Importantly, the effect of learning on our results should be mitigated by the controls that were incorporated, including healthy participants and sham stimulation. Also, significantly more task repetitions could potentially be limited by fatigue, which was not observed in our sample (i.e., no participants asked to take a break when offered).

We have not carried out a full examination of the preprocessing and feature extraction [66]. Finally, we did not include asymmetry index measures for the chronic stroke participants as some authors have [24]. Given the heterogeneity of each chronic stroke participant, the extent to which each of these factors contributes to the need to personalize the training models for each individual should be explored further in future studies. These findings may help lead to the development of a closed-loop device that can auto-detect movement phases and deliver therapeutic stimulation when most beneficial for chronic stroke rehabilitation.

## Data Availability

The data, as well as code, that support the findings of this study are available on request from the lead author.

## Supplementary Materials

The following supporting information can be downloaded at: https://www.mdpi.com/article/10.3390/brainsci15010028/s1: Table S1: Participant demographics and characteristics; Table S2: For Figure 2. Disease state classification. Mean accuracies by stimulation state, algorithm and time period; Table S3: For Figure 3. Hold versus Reach classification. Mean accuracies by disease state, stimulation state, time period, and algorithm; Table S4 for Figure 4. Hold versus Reach classification by frequency band, time period, and algorithm.; Table S5 for Figure 5: Hold versus Reach classification. Mean accuracies by disease state, stimulation state, time period and electrode laterality relative to dominant hand; Supplementary Table S6: Statistical analyses; Supplementary Table S7: Multiple comparison of methods for Hold versus Reach for CS active only (Figure 4). Table S8: Mean training time for each algorithm (excluding the ensemble methods). Table S9: tDCS studies with small participant numbers limited subjects; Table S10: To summarize the outcomes from the literature with alternative approaches in machine learning; Table S11: Effect size calculations for main findings [10,14,27,50,57,58,63,66,67,68,69,70,71,72,73,74,75,76,77,78].

## Abbreviations

ADA, adaptive boost; CS, chronic stroke; DT, decision tree; EEG, electroencephalogram; Global, soft-vote ensemble weighted based on the overall accuracy; Hard, hard-vote ensemble; KNN, k-nearest neighbors; LDA, linear discriminant analysis; LR, logistic regression; Me, soft-vote ensemble with weights chosen based on the mean of multiple base estimators; NB, Naïve Bayes; RF, random forest; Stim, stimulation; tDCS, transcranial direct current stimulation; Train, soft-vote ensemble weighted based on training performance; Uni, soft-vote ensemble weighted equally; VR, virtual reality; XG, XGboost.
