# Supplementary material for: Exploring Machine Learning Classification of Movement Phases in Hemiparetic Stroke Patients: A Controlled EEG-tDCS Study
Source: Brain Sci. 2024 Dec 29;15(1):28. doi: 10.3390/brainsci15010028 (PMC11764431; doi:10.3390/brainsci15010028)
Supplement: Supplementary file 1 [file brainsci-15-00028-s001.zip › brainsci-3357154-supplementary.pdf]

Supplementary Table S1: Participant demographics and characteristics

Group	ID	Sex	Age (Years)	Hand Tested	Stroke Type
Stroke Active	3	F	70	R	Left ischemic stroke - Subcortical
	4	F	34	R	Left ischemic stroke - Cortical
	5	M	67	L	Bilateral ischemic stroke - subcortical
	42	F	62	L	Right ischemic stroke - Subcortical
	43	M	78	R	Left ischemic stroke - Cortical
Stroke Sham	13	M	65	L	Right ischemic stroke - Subcortical
	15	M	60	R	Left ischemic stroke - Cortical
	17	M	47	L	Right ischemic stroke - Subcortical
	18	F	74	L	Right ischemic stroke - Cortical
	21	M	68	L	Right ischemic stroke - Subcortical
Control Active	22	M	32	L	
	24	M	37	R	
	25	F	35	R	
	26	M	29	R	
	29	F	76	R	
	30	F	75	L	
Control Sham	20	M	71	R	
	23	F	31	L	
	27	M	34	R	
	28	F	28	R	
	36	F	50	R	

Supplementary Table S2: For Figure 2. Disease state classification. Mean accuracies by stimulation state, algorithm and time period.

		**Mean Accuracy**	
**Algorithm**	**Time period**	**Active**	**Sham**
ADA	Pre	0.862	0.665
	Intra5	0.805	0.578
	Intra15	0.766	0.611
	Post	0.787	0.548
DT	Pre	0.855	0.68
	Intra5	0.743	0.53
	Intra15	0.71	0.574
	Post	0.72	0.492
Global	Pre	0.893	0.744
	Intra5	0.936	0.711
	Intra15	0.899	0.738
	Post	0.938	0.694
Hard	Pre	0.878	0.73
	Intra5	0.936	0.711
	Intra15	0.899	0.738
	Post	0.938	0.694
KNN	Pre	0.909	0.793
	Intra5	0.792	0.579
	Intra15	0.754	0.614
	Post	0.775	0.548
LDA	Pre	0.633	0.608
	Intra5	0.683	0.504
	Intra15	0.656	0.557

	Post	0.663	0.47
LR	Pre	0.743	0.623
	Intra5	0.747	0.509
	Intra15	0.706	0.561
	Post	0.726	0.475
Me	Pre	0.87	0.719
	Intra5	0.936	0.711
	Intra15	0.899	0.738
	Post	0.938	0.694
NB	Pre	0.68	0.598
	Intra5	0.77	0.555
	Intra15	0.733	0.595
	Post	0.748	0.521
RF	Pre	0.933	0.803
	Intra5	0.789	0.572
	Intra15	0.755	0.61
	Post	0.771	0.539
Train	Pre	0.888	0.756
	Intra5	0.936	0.711
	Intra15	0.899	0.738
	Post	0.938	0.694
Uni	Pre	0.749	0.694
	Intra5	0.936	0.704
	Intra15	0.899	0.711
	Post	0.938	0.738
XG	Pre	0.945	0.694
	Intra5	0.821	0.816

	Intra15	0.783	0.595
	Post	0.806	0.627

Supplementary Table S3: For Figure 3. Hold versus Reach classification. Mean accuracies by disease state, stimulation state, time period, and algorithm.

		Mean Accuracy (%)			
Time period	Algorithm	Group and Stimulation State			
		Chronic Stroke Sham	Chronic Stroke Active	Healthy Control Sham	Healthy Control Active
pre	ADA	0.687	0.722	0.797	0.718
pre	DT	0.685	0.724	0.8	0.72
pre	global	0.687	0.724	0.8	0.719
pre	hard	0.686	0.724	0.8	0.72
pre	KNN	0.685	0.712	0.798	0.704
pre	LDA	0.686	0.724	0.8	0.72
pre	LR	0.685	0.724	0.8	0.72
pre	me	0.686	0.724	0.8	0.72
pre	NB	0.685	0.724	0.8	0.718
pre	RF	0.685	0.711	0.792	0.708
pre	train	0.686	0.724	0.8	0.719
pre	uni	0.685	0.724	0.8	0.72
pre	XG	0.691	0.722	0.794	0.707
intra5	ADA	0.731	0.738	0.8	0.703
intra5	DT	0.737	0.745	0.801	0.704
intra5	global	0.715	0.716	0.794	0.703
intra5	hard	0.715	0.716	0.794	0.703
intra5	KNN	0.73	0.738	0.8	0.703

intra5	LDA	0.743	0.746	0.801	0.704
intra5	LR	0.743	0.746	0.801	0.704
intra5	me	0.715	0.716	0.794	0.703
intra5	NB	0.732	0.739	0.8	0.704
intra5	RF	0.729	0.738	0.8	0.704
intra5	train	0.715	0.716	0.794	0.703
intra5	uni	0.715	0.716	0.794	0.703
intra5	XG	0.729	0.735	0.799	0.703
intra15	ADA	0.716	0.754	0.809	0.746
intra15	DT	0.722	0.761	0.811	0.751
intra15	global	0.705	0.738	0.796	0.729
intra15	hard	0.705	0.738	0.796	0.729
intra15	KNN	0.714	0.753	0.808	0.746
intra15	LDA	0.729	0.761	0.811	0.751
intra15	LR	0.729	0.761	0.812	0.752
intra15	me	0.705	0.738	0.796	0.729
intra15	NB	0.717	0.755	0.809	0.747
intra15	RF	0.714	0.753	0.808	0.747
intra15	train	0.705	0.738	0.796	0.729
intra15	uni	0.705	0.738	0.796	0.729
intra15	XG	0.715	0.752	0.807	0.744
post	ADA	0.764	0.747	0.821	0.722
post	DT	0.77	0.755	0.823	0.726
post	global	0.745	0.727	0.813	0.712
post	Hard	0.745	0.727	0.813	0.712
post	KNN	0.763	0.746	0.82	0.722
post	LDA	0.777	0.755	0.823	0.726

post	LR	0.777	0.755	0.823	0.726
post	Me	0.745	0.727	0.813	0.712
post	NB	0.764	0.748	0.82	0.723
post	RF	0.761	0.746	0.82	0.722
post	Train	0.745	0.727	0.813	0.712
post	Uni	0.745	0.727	0.813	0.712
post	XG	0.762	0.745	0.82	0.721

Supplementary Table S4 for Figure 4. Hold versus Reach classification by frequency band, time period, and algorithm.

		**Mean Accuracy**				
**Time Period**	**Algorithm**	**Frequency Band**				
		**Alpha**	**Beta**	**Delta**	**Gamma**	**Theta**
pre	ADA	0.724	0.723	0.725	0.738	0.721
pre	DT	0.724	0.724	0.724	0.721	0.724
pre	global	0.722	0.721	0.722	0.724	0.722
pre	hard	0.724	0.724	0.724	0.722	0.724
pre	KNN	0.711	0.708	0.713	0.724	0.705
pre	LDA	0.724	0.724	0.724	0.713	0.724
pre	LR	0.724	0.724	0.724	0.724	0.724
pre	me	0.724	0.724	0.724	0.724	0.724
pre	NB	0.724	0.724	0.725	0.724	0.724
pre	RF	0.632	0.636	0.629	0.724	0.624
pre	train	0.724	0.724	0.724	0.648	0.724
pre	uni	0.724	0.724	0.724	0.724	0.724
pre	XG	0.72	0.717	0.716	0.724	0.713
intra5	ADA	0.729	0.727	0.728	0.724	0.729
intra5	DT	0.745	0.745	0.745	0.728	0.745

intra5	global	0.733	0.731	0.737	0.745	0.738
intra5	hard	0.733	0.731	0.737	0.739	0.738
intra5	KNN	0.727	0.724	0.726	0.739	0.726
intra5	LDA	0.745	0.745	0.745	0.725	0.745
intra5	LR	0.745	0.745	0.745	0.745	0.745
intra5	me	0.733	0.731	0.737	0.745	0.738
intra5	NB	0.726	0.723	0.726	0.739	0.726
intra5	RF	0.721	0.718	0.721	0.725	0.721
intra5	train	0.733	0.731	0.737	0.72	0.738
intra5	uni	0.733	0.731	0.737	0.739	0.738
intra5	XG	0.729	0.727	0.729	0.739	0.73
intra15	ADA	0.744	0.743	0.743	0.729	0.743
intra15	DT	0.761	0.761	0.761	0.742	0.761
intra15	global	0.745	0.75	0.752	0.761	0.751
intra15	hard	0.745	0.75	0.752	0.756	0.751
intra15	KNN	0.741	0.74	0.74	0.756	0.741
intra15	LDA	0.761	0.761	0.761	0.739	0.761
intra15	LR	0.761	0.761	0.761	0.761	0.761
intra15	me	0.745	0.75	0.752	0.761	0.751
intra15	NB	0.741	0.74	0.74	0.756	0.739
intra15	RF	0.736	0.734	0.735	0.738	0.734
intra15	train	0.745	0.75	0.752	0.733	0.751
intra15	uni	0.745	0.75	0.752	0.756	0.751
intra15	XG	0.744	0.744	0.744	0.756	0.744
post	ADA	0.737	0.736	0.737	0.744	0.737
post	DT	0.755	0.755	0.755	0.737	0.755
post	global	0.74	0.741	0.747	0.755	0.745

post	hard	0.74	0.741	0.747	0.751	0.745
post	KNN	0.734	0.733	0.734	0.751	0.734
post	LDA	0.755	0.755	0.755	0.734	0.755
post	LR	0.755	0.755	0.755	0.755	0.755
post	me	0.74	0.741	0.747	0.755	0.745
post	NB	0.734	0.733	0.734	0.751	0.734
post	RF	0.729	0.727	0.728	0.733	0.728
post	train	0.74	0.741	0.747	0.727	0.745
post	uni	0.74	0.741	0.747	0.751	0.745
post	XG	0.738	0.737	0.738	0.751	0.738

Supplementary Table S5 for Figure 5: Hold versus Reach classification. Mean accuracies by disease state, stimulation state, time period and electrode laterality relative to dominant hand.

			**Mean Accuracy**			
**Electrode**	**Time period**	**Algorithm**	**Groups**			
			**CS_Sham**	**CS_Active**	**HC_Sham**	**HC_Active**
ipsi	pre	ADA	0.419	0.379	0.781	0.66
ipsi	pre	DT	0.627	0.651	0.8	0.667
ipsi	pre	KNN	0.702	0.698	0.79	0.72
ipsi	pre	LDA	0.677	0.716	0.79	0.713
ipsi	pre	LR	0.685	0.716	0.8	0.72
ipsi	pre	NB	0.355	0.724	0.267	0.34
ipsi	pre	RF	0.661	0.673	0.785	0.704
ipsi	pre	XG	0.637	0.638	0.771	0.693
ipsi	pre	global	0.655	0.709	0.799	0.703
ipsi	pre	hard	0.678	0.713	0.8	0.711
ipsi	pre	me	0.684	0.714	0.8	0.71

ipsi	pre	train	0.701	0.713	0.8	0.711
ipsi	pre	uni	0.71	0.716	0.8	0.714
ipsi	intra5	ADA	0.622	0.658	0.706	0.644
ipsi	intra5	DT	0.72	0.712	0.703	0.698
ipsi	intra5	KNN	0.655	0.705	0.7	0.643
ipsi	intra5	LDA	0.741	0.725	0.684	0.704
ipsi	intra5	LR	0.743	0.726	0.745	0.704
ipsi	intra5	NB	0.644	0.707	0.692	0.63
ipsi	intra5	RF	0.718	0.703	0.714	0.695
ipsi	intra5	XG	0.632	0.656	0.711	0.646
ipsi	intra5	global	0.704	0.638	0.742	0.657
ipsi	intra5	hard	0.704	0.638	0.742	0.657
ipsi	intra5	me	0.704	0.638	0.742	0.657
ipsi	intra5	train	0.704	0.638	0.742	0.657
ipsi	intra5	uni	0.704	0.638	0.742	0.657
ipsi	intra15	ADA	0.626	0.672	0.71	0.67
ipsi	intra15	DT	0.712	0.725	0.722	0.744
ipsi	intra15	KNN	0.645	0.718	0.706	0.672
ipsi	intra15	LDA	0.725	0.756	0.704	0.752
ipsi	intra15	LR	0.729	0.756	0.761	0.752
ipsi	intra15	NB	0.634	0.721	0.695	0.659
ipsi	intra15	RF	0.707	0.715	0.732	0.739
ipsi	intra15	XG	0.631	0.67	0.717	0.674
ipsi	intra15	global	0.666	0.658	0.761	0.705
ipsi	intra15	hard	0.666	0.658	0.761	0.705
ipsi	intra15	me	0.666	0.658	0.761	0.705
ipsi	intra15	train	0.666	0.658	0.761	0.705

ipsi	intra15	uni	0.666	0.658	0.761	0.705
ipsi	post	ADA	0.633	0.666	0.699	0.647
ipsi	post	DT	0.741	0.723	0.699	0.717
ipsi	post	KNN	0.668	0.713	0.691	0.652
ipsi	post	LDA	0.768	0.747	0.672	0.727
ipsi	post	LR	0.777	0.747	0.755	0.726
ipsi	post	NB	0.655	0.714	0.678	0.638
ipsi	post	RF	0.737	0.705	0.713	0.715
ipsi	post	XG	0.641	0.657	0.704	0.652
ipsi	post	global	0.702	0.596	0.745	0.683
ipsi	post	hard	0.702	0.596	0.745	0.683
ipsi	post	me	0.702	0.596	0.745	0.683
ipsi	post	train	0.702	0.596	0.745	0.683
ipsi	post	uni	0.702	0.596	0.745	0.683
nonIpsi	pre	ADA	0.688	0.696	0.795	0.714
nonIpsi	pre	DT	0.689	0.724	0.8	0.72
nonIpsi	pre	KNN	0.684	0.712	0.798	0.703
nonIpsi	pre	LDA	0.686	0.724	0.8	0.72
nonIpsi	pre	LR	0.685	0.724	0.8	0.72
nonIpsi	pre	NB	0.685	0.724	0.8	0.718
nonIpsi	pre	RF	0.685	0.711	0.791	0.707
nonIpsi	pre	XG	0.692	0.713	0.79	0.708
nonIpsi	pre	global	0.687	0.724	0.8	0.719
nonIpsi	pre	hard	0.686	0.724	0.8	0.72
nonIpsi	pre	me	0.685	0.724	0.8	0.72
nonIpsi	pre	train	0.686	0.724	0.8	0.72
nonIpsi	pre	uni	0.685	0.724	0.8	0.72

nonIpsi	intra5	ADA	0.732	0.735	0.8	0.702
nonIpsi	intra5	DT	0.739	0.745	0.801	0.704
nonIpsi	intra5	KNN	0.731	0.738	0.8	0.703
nonIpsi	intra5	LDA	0.743	0.745	0.801	0.704
nonIpsi	intra5	LR	0.743	0.745	0.801	0.704
nonIpsi	intra5	NB	0.733	0.739	0.8	0.704
nonIpsi	intra5	RF	0.73	0.737	0.8	0.704
nonIpsi	intra5	XG	0.73	0.733	0.799	0.702
nonIpsi	intra5	global	0.718	0.722	0.796	0.695
nonIpsi	intra5	hard	0.718	0.722	0.796	0.695
nonIpsi	intra5	me	0.718	0.722	0.796	0.695
nonIpsi	intra5	train	0.718	0.722	0.796	0.695
nonIpsi	intra5	uni	0.718	0.722	0.796	0.695
nonIpsi	intra15	ADA	0.717	0.749	0.809	0.746
nonIpsi	intra15	DT	0.724	0.761	0.811	0.751
nonIpsi	intra15	KNN	0.715	0.753	0.808	0.746
nonIpsi	intra15	LDA	0.729	0.761	0.811	0.751
nonIpsi	intra15	LR	0.729	0.761	0.812	0.752
nonIpsi	intra15	NB	0.718	0.755	0.809	0.747
nonIpsi	intra15	RF	0.715	0.753	0.808	0.746
nonIpsi	intra15	XG	0.715	0.747	0.807	0.744
nonIpsi	intra15	global	0.703	0.732	0.795	0.735
nonIpsi	intra15	hard	0.703	0.732	0.795	0.735
nonIpsi	intra15	me	0.703	0.732	0.795	0.735
nonIpsi	intra15	train	0.703	0.732	0.795	0.735
nonIpsi	intra15	uni	0.703	0.732	0.795	0.735
nonIpsi	post	ADA	0.766	0.743	0.82	0.722

nonIpsi	post	DT	0.773	0.755	0.823	0.726
nonIpsi	post	KNN	0.765	0.746	0.82	0.722
nonIpsi	post	LDA	0.777	0.755	0.823	0.726
nonIpsi	post	LR	0.777	0.755	0.823	0.726
nonIpsi	post	NB	0.766	0.747	0.821	0.723
nonIpsi	post	RF	0.764	0.746	0.82	0.722
nonIpsi	post	XG	0.764	0.74	0.82	0.721
nonIpsi	post	global	0.754	0.721	0.815	0.71
nonIpsi	post	hard	0.754	0.721	0.815	0.71
nonIpsi	post	me	0.754	0.721	0.815	0.71
nonIpsi	post	train	0.754	0.721	0.815	0.71
nonIpsi	post	uni	0.754	0.721	0.815	0.71

Supplementary Table S6: Statistical analyses

**Comparison**	**Test**				
**Fig 2: Classification of Healthy vs Stroke, Active only, over time**	**One-way repeated measures ANOVA**	**DFn**	**DFd**	**F**	**p**
	Effect of time:	3	36	1.42	0.254
	**Mauchly's Sphericity**			**W**	**p**
				0.000106	2.81e-19
	**Greenhouse-Geisser Correction**		**DF[GG]**	**GGe**	**p[GG]**
			12	0.339	0.257
**Classification of Healthy vs.**	**One-way repeated measures ANOVA**	**DFn**	**DFd**	**F**	**p**

**Stroke, Sham only, over time**					
	Effect of time:	3	36	20.3	7.21e-08
	**Mauchly's Sphericity**			**W**	**p**
				0.000182	4.37e-18
	**Greenhouse-Geisser Correction:**		**DF[GG]**	**GGe**	**p[GG]**
			12	0.346	0.000605
	**Individual comparisons**	**df**	**t**	**p**	**Adjusted p (multiple comparisons)**
	pre vs. intra5	24	-3.1	0.005	0.023
	pre vs. intra15	24	-2.1	0.046	0.184
	pre vs. post	23	-3.8	0.000939	0.006
	intra5 vs. intra15	24	1.1	0.271	0.542
	intra5 vs. post	24	0.776	0.446	0.542
	intra15 vs. post	23	1.9	0.072	0.217
**Classification of Healthy vs. Stroke, Active vs Sham over time**	**Two-way repeated measures ANOVA**	**DFn**	**DFd**	**F**	**p**
	Effect of group:	1	24	4.5	0.044
	Effect of time:	3	72	11.1	4.51e-06
	Group – time interaction effect	3	72	9.95	1.45e-05
	**Mauchly's Sphericity**			**W**	**p**
	Effect of time:			0.000353	7.43e-37
	Effect of group – time interaction:			0.000353	7.43e-37
	**Greenhouse-Geisser Correction**		**DF[GG]**	**GGe**	**p[GG]**

	Effect of time:		25	0.342	0.003
	Effect of group – time interaction:		25	0.342	0.004
	**Individual comparisons**	**df**	**t**	**p**	**Adjusted p (Bonferroni)**
	Active vs. sham at pre (normalized)	24	-0.000265	1	1
	Active vs. sham at intra5 (normalized)	24	-3	0.00575	0.023
	Active vs. sham at intra15 (normalized)	24	-1	0.322	1
	Active vs. sham at post (normalized)	24	-3.2	0.00341	0.013
**Fig 3: Movement classification, CS Active only, over time**	**One-way repeated measures ANOVA**	**DFn**	**DFd**	**F**	**p**
	Effect of time:	3	36	39.5	1.76e-11
	**Mauchly's Sphericity**			**W**	**p**
	Effect of time:			1.47e-05	1.18e-23
	**Greenhouse-Geisser Correction**		**DF[GG]**	**GGe**	**p[GG]**
			12	0.335	3.88e-05
	**Individual comparisons**	**df**	**t**	**p**	**Adjusted p (multiple comparisons)**
	Pre vs. intra5	15	2.4	0.028	0.086
	Pre vs. intra15	17	9.2	4.84e-08	2.9e-07
	Pre vs. post	16	5.5	0.000583	0.000292
	Intra5 vs. intra15	22	4.1	0.000496	0.002
	Intra5 vs. Post	24	-2	0.053	0.105

	Intra15 vs. Post	23	2	0.06	0.105
**Movement classification, CS Active vs. CS Sham.**	**Two-way repeated measures ANOVA**	**DFn**	**DFd**	**F**	**p**
	Effect of group:	1	24	20.2	0.000151
	Effect of time:	3	72	2.3e+02	4.45e-37
	Effect of group-time interaction:	3	72	112	5.25e-27
	**Mauchly's Sphericity**			**W**	**p**
	Effect of time:			0.002	1.16e-27
	Effect of group-time interaction:			0.002	1.16e-27
	**Greenhouse-Geisser Correction**		**DF[GG]**	**GGe**	**p[GG]**
	Effect of time:		25	0.347	2.26e-14
	Effect of group-time interaction:		25	0.347	7.54e-11
	**Individual comparisons**	**df**	**t**	**p**	**Adjusted p (multiple comparisons)**
	CS active vs CS sham at pre	14	-26	1.06e-13	4.24e-13
	CS active vs CS sham at intra5	23	-0.915	0.37	1
	CS active vs. CS sham at intra 15	24	-9.7	9.43e-10	3.78e-09
	CS active vs CS sham at post	24	3.8	0.000958	0.00383
**Movement classification, CS**	**Two-way repeated measures ANOVA**	**DFn**	**DFd**	**F**	**p**

**sham vs. HC active.**					
	Effect of group:	1	24	0.4	0.533
	Effect of time:	3	72	203	4.78e-35
	Effect of group-time interaction:	3	72	243	1.33e-37
	**Mauchly's Sphericity**			**W**	**p**
	Effect of time:			0.031	1.55e-15
	Effect of group-time interaction:			0.031	1.55e-15
	**Greenhouse-Geisser Correction**		**DF[GG]**	**GGe**	**p[GG]**
	Effect of time:		36	0.504	8.23e-19
	Effect of group-time interaction:		36	0.504	4.21e-20
	**Individual comparisons**	**df**	**t**	**p**	**Adjusted p (multiple comparisons)**
	CS sham vs HC active at pre	14	-18	6.56e-11	2.62e-10
	CS sham vs HC active at intra5	12	7.8	4.66e-06	1.86e-05
	CS sham vs HC active at intra15	24	-7.3	1.63e-07	6.52e-07
	CS sham vs HC active at post	17	10	5.92e-09	2.37e-08
**Movement classification, CS active vs. HC active.**	**Two-way repeated measures ANOVA**	**DFn**	**DFd**	**F**	**p**
	Effect of group:	1	24	38.4	2.1e-06
	Effect of time:	3	72	103	4.97e-26

	Effect of group-time interaction:	3	72	18	8.28e-09
	**Mauchly's Sphericity**			**W**	**p**
	Effect of time:			0.002	2.52e-29
	Effect of group-time interaction:			0.002	2.52e-29
	**Greenhouse-Geisser Correction**		**DF[GG]**	**GGe**	**p[GG]**
	Effect of time:		35	0.488	7.2e-14
	Effect of group-time interaction:		35	0.488	2.47e-05
	**Individual comparisons**	**df**	**t**	**p**	**Adjusted p (multiple comparisons)**
	CS active vs HC active at pre	23	2.6	0.0162	0.0324
	CS active vs HC active at intra5	12	7.7	5.28e-06	2.11e-05
	CS active vs HC active at intra15	24	2.3	0.0326	0.13
	CS active vs HC active at post	18	6	1.22e-05	4.88e-05
**Comparison of hold vs. reach classification, HC sham vs. CS active**	**Two-way repeated measures ANOVA**	**DFn**	**DFd**	**F**	**p**
	Effect of group:	1	24	0.4	0.533
	Effect of time:	3	72	203	4.78e-35
	Effect of group-time interaction:	3	72	2.4e+02	1.33e-37
	**Mauchly's Sphericity**			**W**	**p**

	Effect of time:			0.031	1.55e-15
	Effect of group-time interaction			0.031	1.55e-15
	**Greenhouse-Geisser Correction**		**DF[GG]**	**GGe**	**p[GG]**
	Effect of time:		36	0.504	8.23e-19
	Effect of group-time interaction		36	0.504	4.21e-20
**Movement classification, CS active vs. HC sham.**	**Two-way repeated measures ANOVA**	**DFn**	**DFd**	**F**	**p**
	Effect of group:	1	24	745	1.39e-19
	Effect of time:	3	72	766	2.8e-22
	Effect of group-time interaction:	3	72	24	8.4e-11
	**Mauchly's Sphericity**			**W**	**p**
	Effect of time:			0.000456	1.26e-35
	Effect of group-time interaction:			0.000456	1.26e-35
	**Greenhouse-Geisser Correction**		**DF[GG]**	**GGe**	**p[GG]**
	Effect of time:		28	0.384	6.1e-10
	Effect of group-time interaction:		28	0.384	2e-05
	**Individual comparisons**	**df**	**t**	**p**	**Adjusted p (multiple comparisons)**
	CS active vs HC sham at pre	20	-51	2.3e-22	9.2e-22
	CS active vs HC sham at intra5	13	-18	8.85e-11	3.54e-10

	CS active vs HC sham at intra15	22	-16	9.81e-14	3.92e-13
	CS active vs HC sham at post	15	-22	6.63e-13	2.65e-12
**Movement classification, HC Active vs. HC Sham.**	**Two-way repeated measures ANOVA**	**DFn**	**DFd**	**F**	**p**
	Effect of group:	1	24	3.4e+03	2.12e-27
	Effect of time:	3	72	109	1.04e-26
	Effect of group-time interaction:	3	72	69.3	3.49e-21
	**Mauchly's Sphericity**			**W**	**p**
	Effect of time:			0.01	6.35e-21
	Effect of group-time interaction:			0.01	6.35e-21
	**Greenhouse-Geisser Correction**		**DF[GG]**	**GGe**	**p[GG]**
	Effect of time:		29	0.404	3.91e-12
	Effect of group-time interaction:		29	0.404	7.03e-10
	**Individual comparisons**	**df**	**t**	**p**	**Adjusted p (multiple comparisons)**
	HC active vs HC sham at intra15	22	19	5.66e-15	2.26e-14
**Fig 4: Comparison of classification methods for Hold vs. Reach, CS Active only (multiple comparison results in**	**One-way ANOVA**	**DFn**	**DFd**	**F**	**p**

**Supplementary Table 7)**					
		6	2.5e+02	6.3	3.41e-06
**Overall accuracy over time, averaged over all frequencies, in CS Active only**	**One-way repeated measures ANOVA**	**DFn**	**DFd**	**F**	**p**
	Effect of time:	3	36	25.7	4.67e-09
	**Mauchly's Sphericity**			**W**	**p**
	Effect of time:			5.09e-07	3.73e-31
	**Greenhouse-Geisser Correction**		**DF[GG]**	**GGe**	**p[GG]**
	Effect of time:		12	0.334	0.000274
	**Individual comparisons**	**df**	**t**	**p**	**Adjusted p (multiple comparisons)**
	Pre vs. Intra 5	14	-2.6	0.022	0.045
	Pre vs. Intra 15	15	-4.6	0.000323	0.002
	Pre vs. post	15	-3.8	0.002	0.007
	Intra5 vs. Intra 15	24	-4.6	0.000104	0.000624
	Intra5 vs. post	24	-2.8	0.011	0.032
	Intra15 vs. post	24	1.8	0.087	0.087
**Comparison of classification accuracy by frequency band over time**	**One-way ANOVA**	**DFn**	**DFd**	**F**	**p**
Delta	Effect of time:	3	48	13.2	2.13e-06
	**Individual comparisons**	**df**	**t**	**p**	**Adjusted p (multiple comparisons)**

	Pre vs intra5	14	2.6	0.022	0.045
	Pre vs intra15	15	-4.5	0.000426	0.002
	Pre vs post	15	-3.8	0.002	0.008
	Intra5 vs intra15	24	-4.6	0.000124	0.000744
	Intra5 vs post	24	-2.8	0.01	0.031
	Intra15 vs post	24	1.7	0.104	0.104
	**One-way ANOVA**	**DFn**	**DFd**	**F**	**p**
Theta	Effect of time:	3	48	12.9	2.62e-06
	**Individual comparisons**	**df**	**t**	**p**	**Adjusted p (multiple comparisons)**
	Pre vs intra5	14	-2.7	0.018	0.053
	Pre vs intra15	14	-4.4	0.00052	0.003
	Pre vs post	14	-3.7	0.002	0.009
	Intra5 vs intra15	24	-4.3	0.000221	0.001
	Intra5 vs post	24	-2.5	0.018	0.053
	Intra15 vs post	24	1.8	0.094	0.094
	**One-way ANOVA**	**DFn**	**DFd**	**F**	**p**
Alpha		3	48	11.8	6.34e-06
	**Individual comparisons**	**df**	**t**	**p**	**Adjusted p (multiple comparisons)**
	Pre vs intra5	14	-2.4	0.03	0.06
	Pre vs intra15	15	-4.3	0.000655	0.03
	Pre vs post	15	-3.5	0.003	0.013
	Intra5 vs intra15	24	-4.5	0.000153	0.000918
	Intra5 vs post	24	-2.6	0.016	0.047
	Intra15 vs post	24	1.8	0.089	0.089

	**One-way ANOVA**	**DFn**	**DFd**	**F**	**p**
Beta		3	48	13.3	1.95e-06
	**Individual comparisons**	**df**	**t**	**p**	**Adjusted p (multiple comparisons)**
	Pre vs intra5	15	-2.3	0.039	0.078
	Pre vs intra15	15	-4.7	0.000292	0.001
	Pre vs post	15	-3.6	0.002	0.01
	Intra5 vs intra15	24	-5.1	3.5e-05	0.00021
	Intra5 vs post	24	-2.9	0.009	0.026
	Intra15 vs post	24	2.2	0.04	0.078
	**One-way ANOVA**	**DFn**	**DFd**	**F**	**p**
Gamma		3	48	16.1	2.25e-07
	**Individual comparisons**	**df**	**t**	**p**	**Adjusted p (multiple comparisons)**
	Pre vs intra5	16	-2.9	0.011	0.03
	Pre vs intra15	17	-5.2	6.34e-05	0.00038
	Pre vs post	17	-4.4	0.000387	0.002
	Intra5 vs intra15	23	-4.3	0.00027	0.001
	Intra5 vs post	24	-2.8	0.01	0.03
	Intra15 vs post	24	1.4	0.173	0.173
**Comparison of classification accuracy by method and band**	**Two-way ANOVA**	**DFn**	**DFd**	**F**	**p**
	Effect of method	6	2.2e+02	5.65	1.72e-05
	Effect of band	4	2.2e+02	0.203	0.936
	Band-method interaction	24	2.2e+02	0.046	1

**Fig 5: Comparison of classification accuracy by electrode laterality, over time, CS sham only**	**Two-way repeated measures ANOVA**	**DFn**	**DFd**	**F**	**p**
	Effect of laterality:	1	24	0.212	0.649
	Effect of time:	3	72	6.3	0.00074
	Effect of laterality-time interaction:	3	72	3.18	0.029
	**Mauchly's Sphericity**			**W**	**p**
	Effect of time:			0.005	3.69e-24
	Effect of laterality-time interaction			0.005	3.69e-24
	**Greenhouse-Geisser Correction**		**DF[GG]**	**GGe**	**p[GG]**
	Effect of time:		25	0.353	0.017
	Effect of laterality-time interaction		25	0.353	0.085
**Comparison of classification accuracy by electrode laterality, over time, CS active only**	**Two-way repeated measures ANOVA**	**DFn**	**DFd**	**F**	**p**
	Effect of laterality:	1	24	1.89	0.182
	Effect of time:	3	72	4.21	0.008
	Effect of laterality-time interaction:	3	72	7.2	0.000272
	**Mauchly's Sphericity**			**W**	**p**
	Effect of time:			0.028	6.28e-16

	Effect of laterality-time interaction			0.028	6.28e-16
	**Greenhouse-Geisser Correction**		**DF[GG]**	**GGe**	**p[GG]**
	Effect of time:		37	0.51	0.032
	Effect of laterality-time interaction		37	0.51	0.005
	**Individual comparisons (two-sample paired t-test)**	**df**	**t**	**p**	**Adjusted p (multiple comparisons)**
	Contra vs. Ipsi at pre	12	2.2	0.052	0.208
	Contra vs. Ipsi at intra5	12	-7	1.41e-05	5.64e-05
	Contra vs. Ipsi at intra15	12	-4.7	0.00051	0.00204
	Contra vs. Ipsi at post	12	5	0.000286	0.0011
**Comparison of classification accuracy by electrode laterality, over time, HC sham only**	**Two-way repeated measures ANOVA**	**DFn**	**DFd**	**F**	**p**
	Effect of laterality:	1	24	0.006	0.94
	Effect of time:	3	72	0.817	0.489
	Effect of laterality-time interaction:	3	72	0.091	0.965
	**Mauchly's Sphericity**			**W**	**p**
	Effect of time:			0.000312	1.87e-37
	Effect of laterality-time interaction			0.000312	1.87e-37
	**Greenhouse-Geisser Correction**		**DF[GG]**	**GGe**	**p[GG]**

	Effect of time:		25	0.342	0.378
	Effect of laterality-time interaction		25	0.342	0.772
**Comparison of classification accuracy by electrode laterality, over time, HC active only**	**Two-way repeated measures ANOVA**	**DFn**	**DFd**	**F**	**p**
	Effect of laterality:	1	24	0.154	0.698
	Effect of time:	3	72	3.18	0.029
	Effect of laterality-time interaction:	3	72	0.043	0.988
	**Mauchly's Sphericity**			**W**	**p**
	Effect of time:			0.000343	5.3e-37
	Effect of laterality-time interaction			0.000343	5.3e-37
	**Greenhouse-Geisser Correction**		**DF[GG]**	**GGe**	**p[GG]**
	Effect of time:		24	0.339	0.087
	Effect of laterality-time interaction		24	0.339	0.842

Supplementary Table S7: Multiple comparison of methods for Hold versus Reach for CS active only (Fig 4).

	Method 1	Method 2	df	t	p	p.adj
1	Bayesian	Boost	50.7	-0.939	0.352	1
	Bayesian	Clustering	29.8	1.04	0.308	1
	Bayesian	Dimensionality Reduction	26.4	-4.47	0.00013	0.002
	Bayesian	Regression	26.4	-4.48	0.00013	0.002

	Bayesian	Tree	43.5	0.868	0.39	1
	Bayesian	Voting	42.9	-4.49	5.22e-05	0.001
2	Boost	Clustering	30.9	1.69	0.102	0.714
	Boost	Dimensionality Reduction	26.8	-3.94	0.000525	0.008
	Boost	Regression	26.8	-3.94	0.000524	0.008
	Boost	Tree	43.6	1.18	0.243	1
	Boost	Voting	84.4	-3.46	0.000852	0.011
3	Clustering	Dimensionality Reduction	36.4	-4.56	5.53e-05	0.001
	Clustering	Regression	36.4	-4.56	5.53e-05	0.001
	Clustering	Tree	51.6	0.341	0.735	1
	Clustering	Voting	25.9	-3.98	0.000495	0.008
4	Dimensionality Reduction	Regression	38	-0.000657	0.999	1
	Dimensionality Reduction	Tree	55.5	3.13	0.003	0.034
	Dimensionality Reduction	Voting	23.4	2.26	0.033	0.314
5	Regression	Tree	55.5	3.13	0.003	0.034
	Regression	Voting	23.4	2.27	0.033	0.314
6	Tree	Voting	41.6	-2.23	0.031	0.314

Supplementary Table S8: Mean training time for each algorithm (excluding the ensemble methods).

**Algorithm**	**LR**	**LDA**	**DT**	**RF**	**NB**	**KNN**	**ADA**	**XG**
Time	00:11.4	00:01.8	02:15.6	00:32.4	00:08.8	00:04.1	00:14.8	01:03.8

Supplementary Table S9: tDCS studies with small participant numbers limited subjects

Authors	Title	N (exp/ctrl)
Biasiucci et al. (2018)[58]	Brain-actuated functional electrical stimulation elicits lasting arm motor recovery after stroke	14/13
Gao et al. (2022)[50]	Repetitive Transcranial Magnetic stimulation of the Brain Region Activated by Motor Imagery Involving a Paretic Wrist and Hand for Upper-Extremity Motor Improvement in Severe Stroke: A Preliminary Study	5/5
Mokienko et al. (2013)[14]	Increased motor cortex excitability during motor imagery in brain-computer interface trained subjects	5/6
Jia et al. (2021)[67]	5 Hz rTMS improves motor-imagery based BCI classification performance	11
Ramos-Murguialday et al. (2013)[27]	Brain-machine interface in chronic stroke rehabilitation: a controlled study	16/16
Epperson et al. (2024)[68]	Characterization of an algorithm for autonomous, closed-loop neuromodulation during motor rehabilitation	32
Morone et al. (2022)[69]	May dual transcranial direct current stimulation enhance the efficacy of robot-assisted therapy for promoting upper limb recovery in chronic stroke	33/33
Bolognini et al. (2011)[57]	Neurophysiological and behavioral effects of tDCS combined with constraint-induced movement therapy in poststroke patients	7/7
Oken et al. (2014)[66]	Brain-computer interface with language model-electroencephalography fusion for locked-in syndrome	9
Chew et al. (2020)[10]	Using Transcranial Direct Current stimulation to Augment the Effect of Motor Imagery-Assisted Brain-Computer Interface Training in Chronic Stroke Patients-Cortical Reorganization Considerations	10/9
Lun et al. (2020)[63]	A Simplified CNN Classification Method for MI-EEG via the Electrode Pairs Signals	10

Supplementary Table S10: To summarize the outcomes from the literature with alternative approaches in machine learning

Authors	Used Method (classifiers)	Overall main outcome with limitation
Watts D, Pulice RF, Reilly J. *et al. (2022)*[70]	support vector machine (SVM), extreme learning machine (ELM), and linear discriminant analysis (LDA)	The authors only aimed to demonstrate the feasibility of using machine learning to identify patients likely to respond to tDCS treatment but did not provide conclusive evidence regarding its application to neural diseases.
Hosseinifard B, Moradi MH, Rostami R. (2013)[71]	K-nearest neighbors (KNN), LDA and logistic regression (LR)	The analytical results may support psychiatrists in diagnosing depression but does not provide conclusive evidence for early detection.
Yuan Q, Zhou W, Li S, Cai D. (2011)[72]	backpropagation (BP) algorithm and SVM	The authors successfully found that ELM, an artificial neural network with a single hidden layer, outperformed in terms of training time and classification accuracy, achieving 96.5% accuracy for interictal and ictal EEG signals, but was not effective for stroke or other conditions.
Yang CY, Chen YZ (2024).[73]	SVM	The authors only limited their study to achieving a mean accuracy (over 90%) with the SVM classifier in distinguishing patients with major depressive disorder (MDD) from healthy control (HC) subjects, but no conclusions could be drawn regarding early detection.
Mumtaz W, Ali SSA, Yasin MAM *et al. (2018)*[74]	SVM, LR and Naïve Bayesian (NB)	The study found that EEG-derived synchronization likelihood (SL) could be a promising method for diagnosing depression, but it did not discuss the application of machine learning algorithms in the early detection of neurological diseases
Maitín AM, García-Tejedor AJ, Muñoz JPR (2020)[75]	SVM, KNN (k being considered as the number of nearest neighbors), LR	The authors discussed the growing field of machine learning techniques in EEG for classifying neurodegenerative disorders, but no conclusions were made about the use of EEG/tDCS for neurological diseases like stroke.
Lee M, Hong Y, An S, *et al. (2023)*[76]	machine learning-based regression models (following previous work using LDA)	It was demonstrated that estimating EEG-based network properties in the acute phase of ischemic stroke using a machine learning model can potentially predict cognitive outcomes; however,

		our study focuses on chronic stroke, which involves more complex dynamics.
Lin WY, Chen H, Tseng YJ, *et al. (2018)*[77]	LR, SVM, random forest (RF)	The outcomes are limited as the use of machine learning to predict rehabilitation treatment results after strokes requires further validation and refinement.
Ramirez Campos MS, McCracken HS, Uribe-Quevedo A, *et al. (2024)*[78]	stochastic gradient descent (SGD), SVM, decision tree (DT), Naive Bayes (NB), KNN, RF, multilayer perceptron (MLP)	The authors only focused on using machine learning applied to EEG to discriminate brain activity during task performance with and without haptic feedback, which is not related to neurological diseases.
Our present study	LR, LDA, DT, NB, KNN, RF, AdaBoost, XGBoos	We successfully studied the use of machine learning algorithms to enhance movement phase classification during active tDCS in chronic stroke participants, aiming to improve quality of life and provide early detection in neurorehabilitation and brain-computer interface applications.

Supplementary Table S11: Effect size calculations for main findings.

Finding	Mean 1	Mean 2	Cohen's d
Disease state classification, pre-stim	Active: 0.834	Sham: 0.711	1.41
Disease state classification, post-stim, normalized	Active: 0.986	Sham: 0.825	1.28
Movement state classification, CS Active, pre vs. intra15	Pre: 0.722	Intra15: 0.753	3.18
Movement state classification, intra15, CS Active vs. CS Sham	Active: 0.753	Sham: 0.715	2.71
Movement state classification,	CS: 0.753	HC: 0.745	0.123

intra15, CS Active vs. HC Active			
Movement state classification by frequency band, Gamma, pre vs. intra5	Pre: 0.717	Intra5: 0.730	0.476
Movement state classification by laterality, CS Active, intra5, contra vs. ipsi	Contra: 0.593	Ipsi: 0.681	1.14
Movement state classification by laterality, CS Active, intra15, contra vs. ipsi	Contra: 0.606	Ipsi: 0.697	1.07
